# Tailoring the Management of Colonic Lipomas: A 10-Year Experience of Surgical and Endoscopic Resection

**DOI:** 10.3390/jpm15110544

**Published:** 2025-11-08

**Authors:** Vincenzo Schiavone, Filippo Carannante, Gennaro Melone, Valentina Miacci, Gianluca Costa, Chiara Taffon, Marco Caricato, Gabriella Teresa Capolupo, Gianluca Mascianà

**Affiliations:** 1 Department of Advanced Biomedical Sciences, University of Naples Federico II, AOU “Federico II”, Via Pansini 5, 80131 Naples, Italy; vincenzo.schiavone@unina.it; 2 UOC Chirurgia Colorettale, Fondazione Policlinico Universitario Campus Bio-Medico di Roma, Via Àlvaro del Portillo 200, 00128 Rome, Italy; g.costa@policlinicocampus.it (G.C.); m.caricato@policlinicocampus.it (M.C.); g.masciana@policlinicocampus.it (G.M.); 3 Department of Colorectal Surgery, Università Campus Bio-Medico di Roma, Via Àlvaro del Portillo 200, 00128 Rome, Italy; gennaro.melone@unicampus.it (G.M.); valentina.miacci@unicampus.it (V.M.); 4 General Surgery, Department of Life Sciences, Health and Health Professions, Link Campus University, 00165 Rome, Italy; 5 UOC Anatomia Patologica, Fondazione Policlinico Universitario Campus Bio-Medico di Roma, Via Àlvaro del Portillo 200, 00128 Rome, Italy; c.taffon@policlinicocampus.it

**Keywords:** colorectal surgery, colonic lipoma, minimally invasive surgery, endoscopy, personalized medicine, precision surgery

## Abstract

**Introduction:** Colonic lipomas (CLs) are benign neoplasms originating from adipose tissue within the gastrointestinal tract. They are often asymptomatic, but in certain cases, patients may present with gastrointestinal bleeding or symptoms related to intestinal obstruction. We report the cases of 18 patients undergoing both surgical and endoscopic resection of colonic lipomas. Given the variability in symptoms, lesion size, and patient demographics, the management of CLs represents a clinical scenario where treatment must be tailored to the individual, aligning with the principles of personalized medicine. This study aims to clarify the clinical and morphological factors guiding treatment selection for colonic lipomas, emphasizing a personalized approach to management. **Materials and Methods:** We retrospectively reviewed a prospectively collected database of 18 patients with histological diagnosis of colon lipoma after both endoscopic and surgical resection at the Campus Bio-Medico University Hospital, Rome, from 2016 to the first months of 2025. **Results:** The average patient age was 66 years, and the average maximum size of lipoma was 2.87 cm. The anatomical location of lipomas is very varied, ranging from the ileocecal valve to the distal sigma, and most procedures were endoscopic. **Conclusions:** Despite the fact that no established guidelines about the management of the CLs are reported in literature, the different approaches are related to symptomatology. Our findings try to clarify and demonstrate how the therapeutic decision, whether endoscopic or surgical, is personalized based on the patients and their clinical condition, illustrating how CL management reflects the broader framework of personalized medicine. Our work confirms that the patients most prone to intussusception phenomena are young women with large colonic lipomas.

## 1. Introduction

Colonic lipomas (CLs) are benign neoplasms originating from adipose tissue within the gastrointestinal tract and are frequently identified incidentally, as they are often asymptomatic. Although their overall incidence remains low, ranging between 0.32% and 4.4% [1] in certain cases, patients may present with gastrointestinal bleeding or symptoms related to intestinal obstruction. In this context, CLs represent the most frequent benign lesion implicated in adult colonic intussusception, which is common in children but infrequent in adults (around 95% of intussusceptions occur in the pediatric population and the remaining 5% occur in adults) [2].

A key diagnostic challenge lies in the differentiation of CLs from malignant colonic tumors at the time of initial evaluation. Several imaging modalities are employed to facilitate diagnosis, including abdominal ultrasound, computed tomography (CT), magnetic resonance imaging (MRI), and endoscopic ultrasonography (EUS). Despite the ability of CT and MRI to characterize the fat nature of these lesions, MRI alone cannot definitively exclude local invasion.

CLs exhibit no significant sex predilection and may arise throughout the gastrointestinal tract, although they predominantly affect the colon. Within the colon, the most frequent sites of involvement include the right colon and cecum (39.6%), followed by the transverse colon (25%) [3]. Symptomatology may include abdominal pain, nausea, vomiting, diarrhea, anemia, or, in some instances, intussusception—a complication that often necessitates surgical intervention. Current recommendations suggest surgical resection for CLs exceeding 2 cm in diameter [4]. Numerous reports in the literature have described cases of giant colonic lipomas leading to intussusception, underscoring the clinical significance of timely diagnosis and management [4,5,6].

The therapeutic management of incidentally detected colonic lipomas remains a matter of debate, with no universally established guidelines. Treatment strategies primarily depend on the patient’s clinical presentation, the lesion’s size, and its anatomical location. Both surgical and endoscopic interventions represent the mainstay of CL management. Endoscopy plays a main role not only in diagnosis but also in the treatment of smaller lesions. Several endoscopic techniques have been developed for the resection of larger, symptomatic colonic lipomas. Among these, lipoma unroofing has emerged as a potentially safer, faster, and technically simpler alternative to more complex procedures such as endoscopic mucosal resection (EMR), dissection-based techniques, or loop-assisted resection [7].

Surgical intervention remains the treatment of choice for large lipomas or for lesions that extend into the muscularis propria or serosal layers. When a definitive preoperative diagnosis is established, local excision through colotomy or segmental colectomy is generally sufficient. Conversely, in cases where the diagnosis is uncertain or complications such as obstruction or intussusception arise, more extensive surgical procedures—including segmental resection, hemicolectomy, or subtotal colectomy—are warranted [3].

A major diagnostic challenge for clinicians is the differentiation of CLs from malignant colonic neoplasms during initial assessment. The clinical, endoscopic, and radiological features of CLs can often mimic those of malignant lesions, making preoperative or pre-resection diagnosis particularly difficult. Indeed, definitive diagnosis ultimately relies on histopathological examination of the excised tissue, which remains the gold standard for confirming the benign nature of these lesions. Given the potential for diagnostic uncertainty, especially in cases where malignancy cannot be confidently excluded, surgical resection with lymphadenectomy is frequently recommended to ensure accurate pathological staging and to guide further oncological management in suspected cases of colonic carcinoma. In this setting, the principles of personalized medicine become increasingly relevant: management decisions must integrate individual patient features (age, sex, and comorbidities), lesion characteristics (size, site, and morphology), and available technical expertise. Unlike standardized “one-size-fits-all” strategies, CL management requires tailoring diagnostic and therapeutic approaches to each patient. In the present study, we report on a series of 18 patients who underwent either surgical or endoscopic resection of colonic lesions that were subsequently confirmed as lipomas on histopathological examination over the past ten years. This cohort illustrates the wide variability in management strategies for what is fundamentally the same pathological entity, emphasizing that patient-specific factors, lesion characteristics, and procedural considerations can substantially influence both the choice of intervention and the overall treatment pathway. By presenting these cases, we aim to highlight how individualized clinical judgment and a multidisciplinary approach are essential in optimizing outcomes and ensuring safe, effective management of colonic lipomas.

## 2. Material and Methods

We retrospectively reviewed a prospectively collected database of patients with histological diagnosis of colon lipoma after both endoscopic and surgical resection. All patients were treated at the Fondazione Policlinico Campus Bio-Medico di Roma, Italy. Demographic, clinical, surgical, and pathological data were prospectively collected and retrospectively reviewed. All endoscopic and surgical interventions were carried out by physicians with substantial expertise in their respective fields, each possessing more than five years of dedicated clinical and procedural experience. This ensured a high level of technical proficiency and adherence to contemporary standards of care, thereby minimizing potential procedural variability.

### 2.1. Data Collection

The surgeons’ team compiled a Microsoft Excel Database designed to capture a wide range of clinically relevant information for each patient. The recorded variables included demographic data such as age and sex, detailed anatomical information regarding the location of the colonic lipoma, and documentation of any associated clinical symptoms, including abdominal pain, gastrointestinal bleeding, or bowel obstruction. In addition, the database included a record of all diagnostic tests performed, encompassing endoscopic evaluations, imaging studies, and any ancillary laboratory investigations. Therapeutic interventions were carefully documented, including both endoscopic and surgical procedures, with detailed notes on the specific technique employed, intraoperative findings, and perioperative outcomes. Definitive histological examination results were also recorded, providing a reliable reference for confirming the benign nature of the lesions and ensuring the accuracy of the dataset.

This retrospective study analyzed 18 cases of colonic lipoma (CL) treated between January 2016 and March 2025. The study protocol was approved by the Regional Review Board (187.25 CET2 cbm), and all patients provided informed consent for data collection and analysis. The structured nature of the database ensured consistency and reliability in data reporting, enabling robust retrospective analysis and supporting evidence-based conclusions regarding both endoscopic and surgical management of colonic lipomas.

### 2.2. Patient Selection

The inclusion criteria for the present study encompassed adult patients who had received a histologically confirmed diagnosis of colonic lipoma and who subsequently underwent either endoscopic or surgical resection at our institution within the defined study period. Patients were included regardless of lesion size, location within the colon, or symptomatology, provided that a definitive histopathological diagnosis was available. Specifically, patients were included even if the lipoma had been incidentally identified during surgery for unrelated gastrointestinal or abdominal pathologies, as these cases were considered relevant to provide a comprehensive overview of the surgical management and presentation spectrum of gastrointestinal lipomas. This approach ensured a comprehensive evaluation of all clinically relevant cases of colonic lipoma managed at our center, allowing for a thorough assessment of demographic, clinical, and procedural variables. Exclusion criteria were established to enhance the internal validity of the study and to ensure that the analyzed cohort accurately reflected patients treated primarily for colonic lipomas. Additionally, cases with incomplete clinical or histological data were excluded to maintain the integrity and reliability of the dataset, as missing information could compromise the accuracy of analyses regarding presentation, management, and outcomes. Finally, lesions located outside the colon, including those in the rectum, small intestine, or other segments of the gastrointestinal tract, were not considered for inclusion, given the study’s specific focus on colonic pathology.

### 2.3. Clinical Management and Decision-Making

All patients included in the study were evaluated within a multidisciplinary framework, involving a collaborative team of colorectal surgeons, gastroenterologists, oncologists, and radiologists. This multidisciplinary approach ensured a comprehensive assessment of each case, integrating expertise from multiple specialties to optimize diagnostic accuracy and therapeutic decision-making. The choice between endoscopic and surgical management was made collectively, taking into careful consideration several key factors, including lesion size, morphology, anatomical location within the colon, the presence and severity of clinical symptoms such as abdominal pain, gastrointestinal bleeding, or bowel obstruction, and radiological findings suggestive of atypical lipomatous tumor or potential liposarcoma. Endoscopic resection was generally favored for lesions that were small, asymptomatic, or pedunculated, given the minimally invasive nature of the procedure and its favorable safety profile. Conversely, surgical resection was recommended for larger lesions (typically exceeding 3 cm in diameter) as well as for sessile or anatomically challenging lipomas, those associated with complications such as intussusception or obstruction, or cases in which preoperative evaluation could not definitively exclude malignancy. This decision-making mode emphasized the importance of individualized patient care, balancing procedural risk, expected clinical benefit, and oncological safety. By integrating clinical, endoscopic, and radiological data within a multidisciplinary discussion, the management strategy aimed to achieve optimal outcomes while minimizing unnecessary invasive interventions.

### 2.4. Endoscopic Techniques

**Figure 1:**
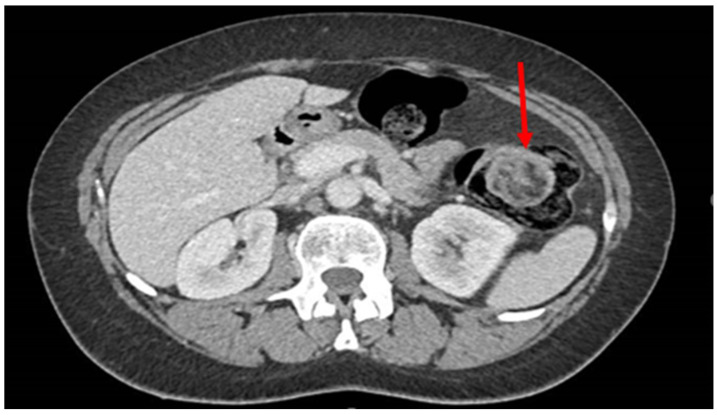
CT scan image of a colon lipoma. CT scan image of a colon lipoma (red arrow).

Endoscopic resections were performed using endoscopic mucosal resection (EMR) for small, pedunculated lipomas, which allows for safe excision of lesions with minimal involvement of the underlying colonic wall. For larger or sessile lesions, loop-assisted resection or the unroofing technique was applied to minimize perforation risk. All endoscopic procedures were performed by experienced gastroenterologists with more than 10 years of expertise in advanced colorectal endoscopy. The procedures were conducted according to established institutional protocols, incorporating careful pre-procedural assessment of lesion morphology and location, use of appropriate endoscopic devices and accessories, and continuous intra-procedural monitoring. Particular attention was paid to post-resection site management, including hemostasis and evaluation of potential residual tissue, to ensure complete excision and to minimize recurrence risk. Representative images of the EMR and loop-assisted procedures are presented in Figure 1.

### 2.5. Surgical Techniques

Surgical approaches included local excision, segmental colectomy, or hemicolectomy, depending on the lipoma’s location and size, as well as the presence of complications such as intussusception or obstruction. Local excision was generally favored for small, easily accessible lesions with minimal involvement of the colonic wall, whereas segmental colectomy or hemicolectomy was indicated for larger or more complex lesions, particularly when they were sessile, located in anatomically challenging regions, or associated with complications that necessitated more extensive resection.

All surgical procedures were performed by colorectal surgeons with over 10 years of experience in minimally invasive and open colorectal surgery. This ensured a high level of technical proficiency, adherence to standardized surgical protocols, and careful intraoperative decision-making tailored to the specific characteristics of each lesion. Perioperative planning included detailed assessment of patient comorbidities, preoperative imaging, and anticipated procedural risks, allowing for optimized surgical outcomes. Postoperative care followed established institutional protocols, with structured monitoring for complications, bowel function recovery, and early mobilization, thereby promoting patient safety and facilitating rapid recovery.

### 2.6. Follow-Up and Outcome Measures

Postoperative and post-endoscopic follow-up data were systematically collected through a combination of scheduled outpatient clinic visits and structured telephone interviews, ensuring comprehensive longitudinal monitoring of all patients included in the study. The follow-up protocol was designed to capture both clinical and functional outcomes, with particular attention to early and late postoperative complications, recurrence of the lesion, and overall bowel function, including changes in frequency, consistency, and continence.

Postoperative and post-endoscopic follow-up data were collected through outpatient visits and telephone interviews. Recorded outcomes included early complications, recurrence, and postoperative bowel function. Early complications included procedure-related adverse events such as bleeding, perforation, or infection, while late complications were monitored for signs of stricture formation or persistent gastrointestinal symptoms. No mortality or recurrence was observed during the follow-up period.

## 3. Results

**Figure 2:**
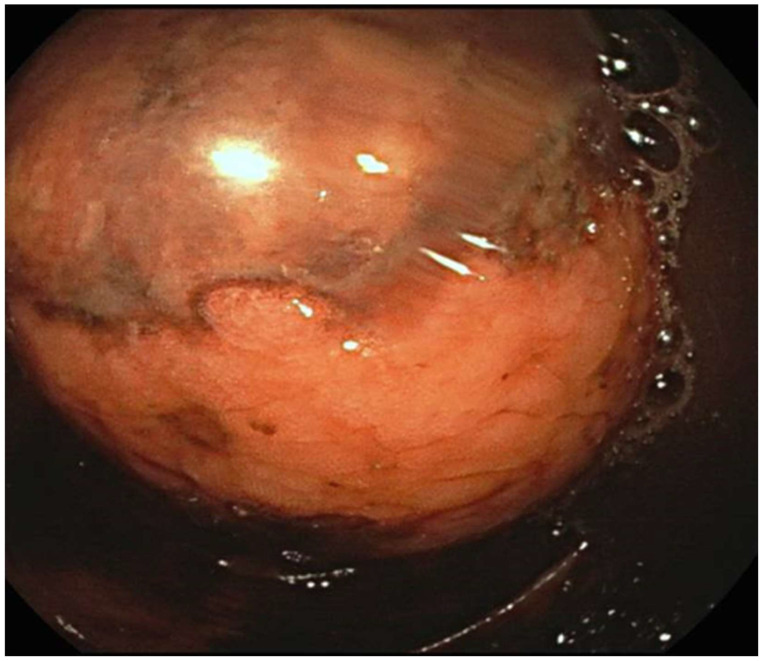
Endoscopic image of a colon lipoma.

**Figure 3:**
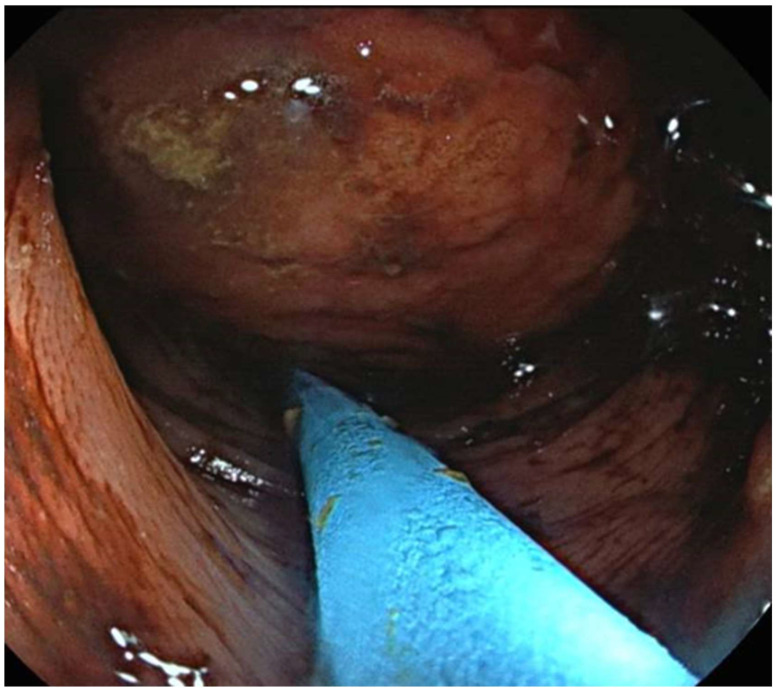
Endoscopic image of a colon lipoma.

We evaluated a total of 18 patients with a histological diagnosis of colonic lipoma related to formations found surgically or endoscopically in different anatomical areas. The period of interest for our research ranges from 2016 to the first months of 2025. The 18 patients analyzed had a variable age, ranging from the youngest (44 years) to the oldest (86 years) with an average age of 66 years. The patients were evenly distributed into 9 males and 9 females. Although the definitive diagnosis was only histological, in most cases colonoscopy (as shown in Figure 2 and Figure 3) was the main pre-treatment diagnostic modality (PTD). In some cases (urgency and/or oncological pathology) there was the possibility of also evaluating the patient with radiological examinations (as shown in Figure 1). Finally, in only 3 cases the presence of the lipomatous formation was unknown before surgery and it was discovered only after the histological examination evaluated on the surgical specimen. We are not aware of any associated symptomatology for all patients (especially those who found the presence of lipoma after a routine colonoscopy), but the most frequently represented symptoms were hematochezia and rectal bleeding. Abdominal pain, on the other hand, was found in patients with occlusion, subocclusion or intussusception who therefore required surgery. From a dimensional point of view, it goes from 6 cm in maximum size (the largest) to 0.5 cm in maximum size (the smallest) with an average of 2.87 cm at histological exam. At the symptomatic level, we can therefore divide our 18 cases into three macro groups:

- 3 completely asymptomatic cases where the presence of colon lipomas was found only after histological examination of the specimen (surgery performed for other reasons).
- 7 cases in which the lipoma was presumably associated with symptoms including abdominal pain, hematochezia, and even intussusception. In 3 of these cases, we had a pre-treatment diagnosis as the symptoms required the execution of pre-endoscopic radiological examinations.
- 8 cases in which the discovery and subsequent removal of the lipoma seem to be accidental, made during routine check-ups or follow-ups (obviously without warning symptoms).

The macroscopic shape mainly found is the polypoid or pseudopolypoid one; there are also some cases of nodular forms and lipomatous fragments. The anatomical location (AL) of lipomas is very varied, ranging from the ileocecal valve to the distal sigma:

- n° 6 in the ascending colon (3 at ileocecal valve);
- n° 6 in the transverse colon;
- n° 1 in the descending colon;
- n° 5 in the sigma.

In 55% of cases the treatment was endoscopic and in the other 45% surgical. Obviously, surgery was reserved for oncological cases or those related to occlusive and/or intussusceptive phenomena. Only one case underwent emergency surgery. There are 2 cases of intussusception, both localized to the transverse colon.

The two cases of intussusception are related to the youngest patients of our group (44 and 51 years old, respectively), both women, and match to the largest formations (6 and 5.5 cm in maximum dimension) (Table 1). The decision on the kind of therapeutic intervention to propose to the patient was made through numerous consultations between surgeons and endoscopists. Obviously, the choice was considered only in non-urgent cases that did not require immediate surgical management. No complications or recurrence are reported in long-term follow-up.

The data about quality of life (measured with the SF-36 instrument) [8] after surgical resection were improved according to ERAS protocols [9].

## 4. Discussion

The management of colonic lipomas (CLs) remains controversial due to their rarity and the lack of standardized guidelines. Although according to the literature they have a range between 0.32% and 4.4% [1], the true prevalence of intraparietal lipomas is almost certainly under-recognized. During colonoscopy, surgery or autopsy, they are generally discovered incidentally. Most cases are asymptomatic, with a small tumor size, and do not need any special treatment. We know from literature that colonic lipomas measuring <2 cm are usually asymptomatic, whereas those >4 cm are symptomatic in 75% of cases [10]. Bleeding from colonic lipomas is an uncommon but recognized complication. Proposed mechanisms include mucosal ulceration overlying the lipoma, pressure necrosis, or erosion into submucosal vessels, all of which can result in significant hemorrhage [11]. They may mimic cancer, depending on multiple factors including tumor size, location, and complications, and often present with moderate hematochezia [12,13]. The most minimally invasive treatment is certainly the endoscopic one. Obviously, its applicability is linked to the dimensional factors of the lipoma and the patient’s clinical situation. Many endoscopic techniques can be used, but Bronswijk et al. (2020) suggested that in patients with large colon lipomas, endoscopic treatment by unroofing, dissection-based resection, EMR and loop-assisted resection provided similar clinical remission rates. However, they concluded that the most optimal resection technique should rely on local expertise and the patient profile [7]. This reinforces the concept that therapeutic decisions in CLs are not uniform but individualized, resonating with the paradigm of personalized medicine, where treatment is adapted to the specific patient context. Even considering the benign nature of the lesion, as we have seen, colonic lipoma can cause intussusceptions that require urgent surgical treatment. Many case reports are present in the literature, but Menegon Tasselli et al. defined the clinical and demographic characteristics of the population that most frequently encounters this outcome. They suggested that colo-colonic intussusception caused by lipoma is more frequent in women, occurring between 40 and 70 years of age, and that the most common location of intussusception is the transverse colon [14]. In fact, Colonic Lipomas are mainly found in women between 50 and 60 years old [15]. In our experience, despite the limited sample, we found an equal distribution of cases between the two sexes. However, we also verified in our cases that the intussusceptive tendency is more represented in young women and involves the transverse colon as per literature. In our case series, lipomas were found as a solitary lesion, although in literature just under 10% are multiple [16]. In 90% of cases, these lesions are localized in the intestinal submucosa, but occasionally they extend into the muscularis propria, while up to 10% are sub-serosal [17]. We found only cases of bleeding or occlusion, although some papers also report the possibility of intestinal perforation caused by lipoma. Cillara et al. (2022) trace the reason for such an eventuality, considering that adipose tissue is relatively avascular and therefore the mucosa overlying the lipomatous lesions of the colon is subject to ischemia and necrosis, ultimately leading to perforation [18]. We also add that Caliskan et al. (2018) told us that intramucosal lipomas also appear to exist incidentally or sporadically, unrelated to Cowden syndrome, occurring in about two-thirds of patients [19]. Proper pathologic and clinical genetic diagnosis of this syndrome, complemented by PTEN germline testing, enables improved cancer prevention through targeted cancer surveillance for these patients and their families. This suggests that genetic profiling and risk stratification may, in selected cases, expand the integration of CL management into precision medicine strategies, linking benign colorectal lesions to broader preventive oncology frameworks.

The main strength of this study is the prospective collection of data over a 10-year period in a single academic institution, which ensured homogeneous management and detailed clinical records. Another strength lies in the dual perspective provided, since both endoscopic and surgical treatments were analyzed, highlighting the spectrum of possible approaches and their tailoring to patient-specific features, consistent with the principles of personalized medicine.

However, several limitations should be acknowledged. First, the relatively small sample size (18 patients) limits the generalizability of our findings. Second, the retrospective nature of the analysis may have introduced selection bias, particularly regarding the decision-making process between surgical and endoscopic resection. Third, the lack of long-term follow-up prevents us from assessing recurrence rates, late complications and post-resection quality of life data. Selection bias represents a possible limitation, as treatment decisions were made by the attending multidisciplinary team based on individualized clinical judgment rather than a standardized protocol. Nonetheless, this reflects real-world practice and reinforces the principle of personalized medicine in colonic lipoma management.

Although our study is limited by the small sample size and single-center analysis, the detailed clinical characterization, procedural description, and inclusion of follow-up outcomes enhance its clinical relevance. Future research should aim to validate these findings prospectively, identify genetic or molecular markers associated with high-risk lipomas, and explore patient-reported outcomes related to post-resection quality of life.

## 5. Conclusions

The main limitation of this study is the number of patients analyzed; however, this must be interpreted considering the epidemiological characteristics of the disease, whose true incidence and prevalence remain largely undefined in the general population. The rarity of reported cases and the frequent incidental nature of diagnosis significantly limit the possibility of collecting large and homogeneous cohorts. Our work confirms the typologies of patients who suffer the consequences of intussusception. However, considering how little is known about the pathology, although benign, we encourage the application of screening programs and routine checks. Tracking down the lesion before it reaches dimensions such as to occlude or cause intussusception could avoid surgical resections in the patient. In the current literature, no standardized or universally accepted guidelines for the management of colonic lipomas are available. Reported approaches are largely dictated by symptomatology, lesion size, and location, leading to significant heterogeneity in clinical practice. In this sense, our findings contribute to the broader discussion on how benign colorectal diseases can be approached through the lens of personalized medicine, ensuring the right treatment for the right patient at the right time. Further and future studies will be useful to define even better if there is a population at risk and if in some patients there is also a possible genetic basis.

## Figures and Tables

**Table 1:** Patients’ Sex, Age, CL PTD, CL size, CL AL, and Treatment Emergency.

Case 1	M	71	colonoscopy	3 cm	sigma	Surgical	0
Case 2	M	51	colonoscopy	3.5 cm	sigma	Endoscopic	0
Case 3	M	74	colonoscopy	1.2 cm	ascending colon	Endoscopic	0
Case 4	F	51	TC-colonoscopy	6 cm	descending colon	Surgical	1
Case 5	F	72	colonoscopy	2.2 cm	ascending colon	Endoscopic	0
Case 6	M	61	TC-RMN-colonoscopy	5 cm	sigma	Surgical	0
Case 7	F	60	colonoscopy	5.5 cm	transverse colon	Endoscopic	0
Case 8	F	65	colonoscopy	0.2 cm	transverse colon	Endoscopic	0
Case 9	M	84		4.5 cm	ascending colon	Surgical	0
Case 10	F	72	colonoscopy	3 cm	transverse colon	Surgical	0
Case 11	M	72	colonoscopy	1.5 cm	sigma	Endoscopic	0
Case 12	F	71	colonoscopy	1.3 cm	ascending colon	Endoscopic	0
Case 13	M	56	colonoscopy	0.5 cm	sigma	Endoscopic	0
Case 14	F	80		2 cm		Surgical	0
Case 15	M	86		1.3 cm	ascending colon	Surgical	0
Case 16	M	51	colonoscopy	0.07 cm	descending colon	Endoscopic	0
Case 17	F	74	colonoscopy	3 cm	ascending colon	Endoscopic	0
Case 18	F	44	TC-colonoscopy	5.5 cm	transverse colon	Surgical	0

## Data Availability

The data presented in this study are available on request from the corresponding author.
